# Tetralogy of Fallot: The Burden of Pulmonary Atresia in the NICU Set-Up: Two Case Reports and a Literature Review

**DOI:** 10.3390/children12060780

**Published:** 2025-06-14

**Authors:** Ion Dragomir, Diana Iulia Vasilescu, Adriana Mihaela Dan, Diana Voicu, Sorin Liviu Vasilescu, Laura Andreea Stefan, Alin Nicolescu, Monica Mihaela Cîrstoiu

**Affiliations:** 1 Department of Neonatology, Emergency University Hospital Bucharest, 050098 Bucharest, Romania; i.dragomirion@gmail.com (I.D.); adriana.dan@umfcd.ro (A.M.D.); 2 “Carol Davila” University of Medicine and Pharmacy Bucharest, 050474 Bucharest, Romania; diana.voicu@umfcd.ro (D.V.); laura-andreea.stefan022025@rez.umfcd.ro (L.A.S.); monica.cirstoiu@umfcd.ro (M.M.C.); 3 Department of Obstetrics and Gynecology, Emergency University Hospital Bucharest, 050098 Bucharest, Romania; drsorinvasilescu@gmail.com; 4 Department of Neonatology, Marie Curie Children’s Hospital, 050098 Bucharest, Romania; 5 Department of Pediatric Cardiology, Marie Curie Children’s Hospital, 050098 Bucharest, Romania; nicolescu_a@yahoo.com

**Keywords:** tetralogy of Fallot, pulmonary atresia, right ventricular outflow tract obstruction, congenital heart disease, mechanical ventilation, hemodynamics, point-of-care ultrasonography

## Abstract

Tetralogy of Fallot (ToF) with pulmonary atresia (PA) and complete right ventricular outflow tract obstruction (RVOTO) represents one of the most critical forms of congenital heart disease in neonates. These cases require complex and timely interventions to ensure survival and optimize long-term outcomes. While surgical correction offers a favorable prognosis, the period from birth to surgery is often marked by significant hemodynamic, respiratory and nutritional challenges, particularly in neonatal intensive care units (NICUs). This study aims to outline a structured, physiology-guided approach to the preoperative management of neonates with ToF and complete RVOTO, emphasizing stabilization strategies, hemodynamic support, ventilatory management and nutritional optimization. We performed a focused literature review of practices in neonatal ToF management and illustrated our experience through two case reports highlighting divergent outcomes in infants with the same anatomical diagnosis. The management strategies covered include delivery room stabilization, the use of prostaglandins, mechanical ventilation techniques, nutritional interventions and the timing of surgical intervention. A phased, physiology-guided management strategy is the key to the successful preoperative treatment of ToF with pulmonary atresia. Optimizing hemodynamics, ensuring adequate pulmonary blood flow and supporting nutritional needs are the main drivers for growth and may reduce the time from diagnosis to surgical correction.

## 1. Introduction

Tetralogy of Fallot (ToF) remains one of the most important congenital heart diseases. A systematic review ranked ToF as the fifth most common, with a prevalence of 34 per 100,000 live births, accounting for up to 10% of all congenital cardiac malformations. It is the most encountered cyanotic congenital heart disease with a prevalence of 1 in 3500 live births [1].

ToF has a multifactorial etiology consisting of genetic and nongenetic factors. In terms of genetic etiology, about 25% of the total cases of TOF have an identifiable chromosomal anomaly [2]. The nongenetic causes of the disease include infections, medication, pregestational diabetes and environmental exposure [3].

The four anatomical characteristics of ToF are the following: ventricular septal defect (VSD), overriding aorta, right ventricular outflow tract obstruction (RVOTO) and right ventricular (RV) hypertrophy. The degree of RVOTO remains the most important feature in the initial steps of the management of a newborn with ToF.

Although the overall survival rate for all patients with ToF after surgical repair remains remarkably high—94.5% at 25 years [4]—the survival rate for those with TOF with pulmonary atresia (PA) is noted at 78% with a rate of rehospitalization that is 4 times higher than that of those with nonPA-ToF [5].

The aim of this article is to adress preoperative management strategies for the subgroup of patients with ToF PA based on our neonatal intensive care unit (NICU) experience and recent data from the literature. For the literature review we used original articles, reviews and guidelines from the PubMed, PubMed Central and Google Scholar databases. The terms used for advanced searching were “tetralogy of Fallot”, “congenital heart disease”, “pulmonary atresia” and “right ventricular outflow tract obstruction”. We only included articles that were published in English, as listed in the Reference Section. To obtain a good understanding of the therapeutic strategy and management checkpoints illustrated in the literature review, we present two cases of ToF PA and complete RVOTO, with the same pathology and different outcomes.

## 2. Preoperative Management

When managing a neonate with a prenatal diagnosis or suspected ToF, it is necessary to assess the grade of RVOTO, as highlighted before. Nowadays most of the newborns we encounter with ToF are prenatally diagnosed with this specific cardiac condition, and fetal ultrasound has demonstrated up to 97% accuracy in diagnosing ToF [6]. The following section outlines a structured approach to the preoperative management of neonates with an antenatal ToF suspicion.

### 2.1. Delivery Room Stabilization

The Neonatal Resuscitation Program (NRP) was created for all newborns that require care in the delivery room (DR), with no particular interventions for newborns with congenital heart disease (CHD). At least two trained NRP providers should be present during high-risk deliveries [7].

The majority of newborns with CHD do not require any resuscitative efforts to fulfill a complete transition, and in the case of resuscitation, it is not necessary to use the NRP. However, it is important to consider that the preductal SpO_2_ targets for newborns with CHD andintracardiac mixing are lower than normal [8].

For newborns with antenatal ToF suspicion, the first steps of the NRP include positioning, drying and stimulations to maintain ventilation and circulation. If the initial resuscitation measures fail to stabilize the infant, respiratory support with positive pressure ventilation is required. For newborns who need positive pressure ventilation, the corrective steps of ventilation when the heart rate fails to increase or ventilation is not maintained are sufficient for stabilization, and less than 0.1% of cases require chest compressions, vascular access and epinephrine [9]. If vascular access is required to administer iv prostaglandin, it might be delayed until NICU admission because it is unlikely that the ductus arteriosus (DA) will close right after delivery.

It should be noted that in the rare case of a newborn with ToF with an absent pulmonary valve, postnatal hypoxia is not a result of decreased pulmonary blood flow (PBF) but of tracheobronchial compressions of the dilated pulmonary branches and lung hypoplasia due to an enlarged RV [10]. In most of these cases, the DA is congenitally absent. Therefore, the adaptation of the NRP is recommended with prone positioning in mild respiratory distress and rapid intubation and mechanical ventilation in severe respiratory distress. In assisted mechanical ventilation, one must be alert when setting the peak pressure and inspiratory time, regarding the risk of air trapping and possible lung hypoplasia. RV dysfunction might be present, especially if pulmonary vascular resistance is high, and in this case, ventilation with high FiO2 and inhaled nitric oxide (iNO) levels should be considered if severe hypoxia is present. It is recommended that PGE infusion should not be initiated [8].

In Table 1, we summarize the main interventions conducted in delivery rooms to achieve a good fetal to postnatal transition.

### 2.2. Ductal-Dependent Circulation

After initial stabilization, NICU admission and an ultrasound confirmation of diagnosis, an intravenous line, preferably a multiple-lumen central venous line, should be inserted, and intravenous prostaglandin E1 infusion should be started in order to maintain the patency of the ductus arteriosus and maintain PBF. TOF with complete RVOTO follows the physiology of the single ventricle/complete intracardiac mixing of congenital heart disease, where the only source for PBF is the PDA. The aortic circulation will be divided between systemic and lung circulation. Systemic flow consists of pulmonary venous return (SpO2 > 95%) and systemic venous return (SpO2 ~70%). Therefore, the SpO2 targets should be set at 75–85%. If the SpO2 levels drop below 75%, oxygen supplementation should not be withheld. Also, higher saturations, e.g., SpO2 > 85%, might be indicative of lower pulmonary vascular resistance (PVR) and pulmonary overcirculation [12].

In settings where a conventional cardiac ultrasound evaluation is impractical or unavailable at the moment [13], we recommend using point-of-care ultrasound (POCUS) evaluation if a trained specialist is available, before and after initiating PGE1 infusion, to identify the cardiac condition and also evaluate the dimensions of the PDA and blood mixing [14]. If severe hypoxia is noted or POCUS evaluation reveals a small PDA, PGE1 infusion should be started using higher doses (0.05–0.1 mcg/kg/min), and the infusion dose should be titrated accordingly to achieve an optimal SpO2 level and DA patency. The maximum recommended infusion dose is 0.4 mcg/kg/min, although higher infusion rates do not produce greater effects and also have a higher risk of adverse reactions [15]. The maintenance dose may be as low as 0.005 mcg/kg/min [16].

There are numerous reports that demonstrate adverse reactions, such as fever, apnea, irritability, rash, convulsions and hypokalemia [17], increase with higher doses and with a longer duration of administration [18]. Based on clinical experience in our center, routine intubation for Alprostadil infusion is not needed; apnea can be managed with caffeine and non-invasive respiratory support [19,20].

The main goal of a neonatologist is to obtain clinical stabilization using the lowest possible doses of Alprostadil in order to minimize adverse events. It is essential to consider that targeting normal SpO2 ranges for a critical care patient with congenital heart disease is not a requirement. In most cases, a rather “normal” SpO2 level (over >95%) in RVOTO is a marker of pulmonary overcirculation, and in the long run, pulmonary edema and cardiac disfunction may develop.

An alternative to PGE1 infusion might be Milrinone infusion to maintain DA patency. Its potential benefits could consist of its inotropic and lusitropic effects and pulmonary vasodilatory action. In one case report, an infusion dose of 0.375 mcg/kg/min was able to open the DA and maintain its patency [21,22]. Although there are no randomized trials that evaluate the efficacy of Milrinone infusion in DA patency, because PGE1 is the standard of care, Milrinone administration may be considered in TOF with RVOTO with severe hypoxia or heart failure under PGE1 infusion. Milrinone’s longer half-life (3 to 10 h in infants) [23] could be a problem in unstable patients that need rapid adjustments. In contrast, PGE1’s half-life is 5 to 15 min [24].

In severe cases when PGE1 infusion is considered to be necessary for a long period of time, stenting the DA via cardiac catheterization might be an option. Also, a palliative intervention such as Blalock–Taussig (BT) shunt may be performed to improve PBF [25,26].

### 2.3. Hemodynamic Considerations

Understanding the variation in systemic vascular resistance (SVR) and pulmonary vascular resistance (PVR) is a key factor in the management of ToF with complete RVOTO. In cases where the SVR is lower due to vasodilation, sepsis being the most common example, a reduction in the left-to-right shunt is noticed, and PBF gradually decreases according to SVR [27]. In these cases, and in severe hypoxic forms of RVOTO with low blood pressure (BP), we support the use of a vasopressor agent such as low doses of norepinephrine (0.05–0.5 mcg/kg/min) with titration according to arterial pressure and clinical stabilization. Vasopressin could be a good alternative [28]. The literature highlights that low doses of norepinephrine do not increase PVR [29].

For an appropriate measurement of BP, an arterial line and invasive continuous monitoring are the gold standard for critically ill neonates [30,31,32].

To prevent the most common adverse event associated with umbilical lines—malposition—the POCUS-guided insertion and verification of the catheter tip can improve the successful rate of insertion and reduce the risk of malposition [32,33,34].

One downside in PA cases where the blood flow is shunted via the PDA is that an arterial line, especially a high-positioned UAC, might reveal low systolic and diastolic BP. In some cases, postductal blood flow is compromised, for example, but not limited at a large PDA secondary to a high dose of PGE1 and pulmonary overcirculation [35].

In ToF PA, elevated PVR functionally reduces PBF, resulting in a hypoxic state that promotes pulmonary vasoconstriction [36]. This vicious circle ends with higher PVR [37], with the direct consequence being hypoxic failure. If PGE1, ventilation and vasopressors fail to improve oxygenation, iNO may reduce PVR and increase PBF. Although the primary indication for iNO in neonates is persistent pulmonary hypertension (PPHN) [38], iNO is used to modulate PVR where other resources have been used up [26], and POCUS cardiac evaluation reveals predominant pulmonary-to-systemic shunt via the PDA. We also use iNO in TOF complicated with overlapping PPHN documented by cardiac ultrasound. We recommend a starting dose of 20 ppm, following the same guide for PPHN [38], and titrate it by maintaining the SpO2 level within the specified ranges, and a POCUS evaluation of the ductal flow reveals mixing circulation via the PDA with predominant systemic-to-pulmonary shunt. If there is no clinical response within 30 min, the dose may be increased to 40 ppm [38] for another 30 min. If no clinical improvement is noticed, iNO may be discontinued [38].

### 2.4. Ventilatory Management

There is no specific data to help guide clinicians in the ventilatory management of newborns with ToF and RVOTO. We suggest a phased approach closely following the physiology of the disease.

Heart–lung interactions, especially with PPV, significantly affect hemodynamics and impact treatment. An increase in intrathoracic pressure affects the venous return and therefore half of the systemic blood flow. The higher the intrathoracic pressure, the lower the venous return. PPV also impacts left ventricular afterload by creating a difference between the intrathoracic and extrathoracic arterial system, generating a “waterfall” effect. The mechanic compression of the alveoli may increase PVR and further limit PBF [39].

In ToF, respiratory muscle oxygen consumption rises with an increasing work of breathing (WOB); therefore when respiratory distress is present, non-invasive ventilation should be considered to lower oxygen demand.

When invasive mechanical ventilation is needed, most neonatal ventilators are time-cycled and pressure-limited and, in the modern era, volume-targeted. When ventilating ToF neonates, the focus is on using the lowest effective settings to control SpO2, the work of breathing and PaCO2 while avoiding overdistension and hyperoxia.

If volume-targeted ventilation is started, the initial set-up of the tidal volume (V_T_) should be 4.5–5.5 mL/kg, considering that this range is intended as a recommendation and may require individual adjustment [40]. The aim is to use the lowest mean airway pressure that minimizes barotrauma and volutrauma, achieves adequate ventilation, optimizes the partial pressure of oxygen (PaO2) and minimizes oxygen toxicity.

Profound sedation may not be a good standard because higher parameters might be needed when it is used. When the patient triggers a spontaneous breath, the intrathoracic pressure drops before PPV is delivered, therefore lowering the PVR [41].

Table 2 summarizes the initial set-up and subsequent adjustment of mechanical ventilation in the NICU.

### 2.5. Preoperative Nutritional Management

Newborns with congenital heart disease are a particular group of patients with high nutritional demands. In some specific types of cardiac lesions, these neonates need nutritional interventions in order to improve their nutritional status and obtain linear growth.

ToF is among the congenital heart diseases that carry a high nutritional risk [42]. In order to obtain optimal growth, ToF patients must have a high energy intake (120–150 kcal/kg/day) and high protein intake (up to 4 g/kg/day).

In clinical practice, high efforts are made to achieve these standards. CHD patients need parenteral nutrition in order to maintain active growth, and in most cases, it is the only acceptable route of nutritional support. Splitting the systemic blood flow can affect the mesenteric perfusion; thus, delaying full enteral nutrition is common in complete RVOTO.

Although breastfeeding remains the recommended nutritional approach in newborns, patients with PA frequently develop hemodynamic instability or respiratory distress or may become fatigued during oral feeding. In most cases, nasogastric tube (NGT) feeding is indicated, with human milk, pasteurized donor human milk or enteral formula being used for infants.

Human milk is the preferred feeding for all infants. Evidence supports the fact that human milk feeding reduces the risk of necrotizing enterocolitis and late-onset sepsis and favors oral feeding rates. Using human milk fortifiers for infants with CHD to meet high energy demands is common practice among these patients, although sufficient data is not available. In instances where human milk or pasteurized donor human milk is not available, infant formula might be used. If standard formula feeding volumes fail to support weight gain, a 1 kcal/mL hypercaloric formula is recommended [43].

If there are no contraindications (e.g., hemodynamic instability), intestinal priming with human milk is indicated from the first days of life. Total enteral volume should be advanced with 10–20 mL/kg/day to a maximum volume of 165 mL/kg/day. It is important to highlight that prostaglandin infusion is not a contraindication of intestinal priming [42].

Postnatally, as PVR falls, systemic blood may shunt through the PDA, reducing distal perfusion. In situations where POCUS evaluation finds significant retrograde flow in the postductal aorta, our current practice is to temporarily stop enteral feeding and adjust PGE1 infusion. The NGT feeds may be reinitiated after the resolution of this diastolic “steal”.

The main goal in CHD patient nutrition is to achieve an optimal weight gain and maintain an optimal nutritional status before surgical intervention. In order to meet these goals, nutritional protocols that are standardized per center are recommended by the current guidelines [43].

## 3. Operative Timing

Although the current literature concludes that center experiences and preferences may be reasonable drivers for the initial treatment strategy, future randomized control trials are needed to stabilize the timing and correction method used [44].

The current literature finds that compared to infants weighing over 2500 g, infants weighing less than 2500 g have a higher risk of mortality following systemic-to-pulmonary artery shunt, a palliative procedure that is used until the complete repair of ToF with pulmonary valve stenosis/atresia [45].

The most common intervention for staged repair is modified BT shunt with or without complete repair a second time [46].

PDA stenting as per the catheter procedure is also feasible and may be performed for those under 2500 g, followed by a planned reintervention for complete repair while waiting for weight gain or hemodynamic stability [47].

## 4. Case Reports

Case 1:

A female newborn weighing 2190 g was delivered spontaneously at 37 weeks and 6 days of gestation, from a twin pregnancy, with an antenatal diagnosis of ToF and intrauterine growth restriction. Apgar scores were 8 at 1 min and 5 min. The infant was admitted to the NICU immediately after birth for monitoring and further investigations.

A POCUS evaluation was performed at 15 min of life, confirming the diagnosis of ToF and complete RVOTO. The DA was not visualized, but two small aortopulmonary collaterals were present.

Initial preductal saturation was 60% without O_2_ supplementation, HR 161 bpm, non-invasive BP 93/53/71 mmHg. Central venous access was obtained, and continuous PGE1 infusion was started at 30 min of life, without clinical improvement. Non-invasive respiratory support was started at 40 min of life. The patient’s clinical condition continued to deteriorate; therefore orotracheal intubation and mechanical ventilation (volume-targeted, pressure support SIMV) were initiated.

**Figure 1:**
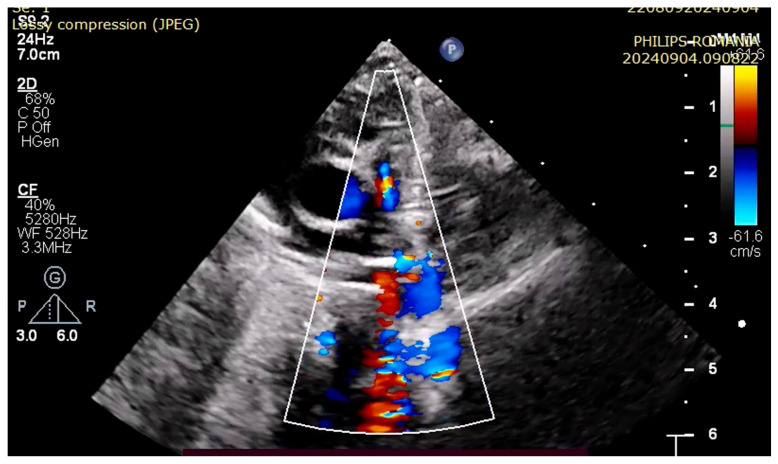
Filiform anterograde flow via pulmonary artery.

**Figure 2:**
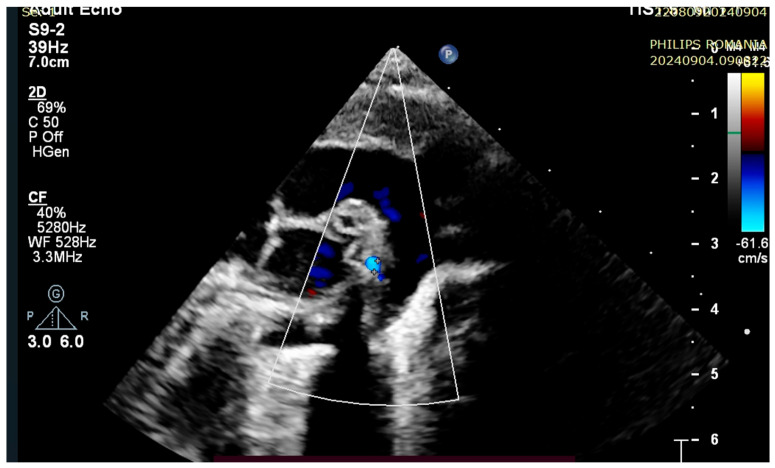
Small aortopulmonary collateral, 1.8 mm.

**Figure 3:**
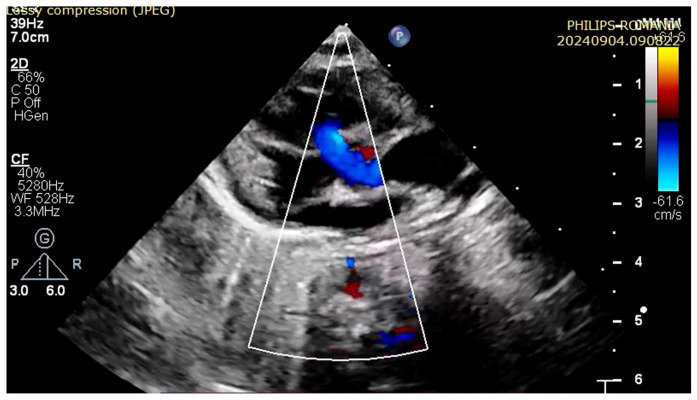
Ventricular septal defect. Overriding aorta.

At 3 h of life, the patient was ultrasound-evaluated by a pediatric cardiologist with the following findings: severe hypoplasia of the trunk and branches of the pulmonary artery, thickened pulmonary valve with very limited opening with filiform anterograde flow, right-sided aorta 30%, wide subaortic ventricular septal defect, left aortic arch, the ductus arteriosus is not visualized, and small collaterals of approximately 1.8 mm from the level of the aortic arch are visualized (Figure 1, Figure 2 and Figure 3). Both the clinical and cardiological evaluations were consistent with iNO administration.

After 30 min of iNO therapy, with PGE1 being up to the maximum infusion rate and with adapted ventilatory settings, there was no significant improvement. Clinical instability did not allow for transfer to a surgical center to perform aortopulmonary shunt. Consequently, Milrinone infusion was started, with SpO2 raising up to 75%.

During this period, the HR and BP were stable, but SpO2 continued to drop, and arterial blood gases revealed metabolic acidosis. In the second day of life, the patient’s condition deteriorated with severe hypoxia, hypotension and severe metabolic acidosis. The continuous infusion of norepinephrine and NaHCO_3_ was added to therapy as an attempt to improve BP. Unfortunately, at the end of the second day of life, the patient died.

Case 2:

A female newborn weighing 1000 g was delivered through emergency Cesarean section for the prolonged rupture of membranes at 32 weeks of gestation, from a twin pregnancy, with an antenatal diagnosis of ToF.

The infant required resuscitation at birth (PPV followed by CPAP). Apgar scores were 7 at 1 min and 9 at 5 min. After delivery room stabilization, the infant was admitted to the NICU for monitoring and further investigations.

A POCUS evaluation was performed at 20 min of life, confirming the diagnosis of ToF and complete RVOTO. The DA was visualized with a 2 mm diameter and bidirectional flow.

Initial preductal saturation was 85% with nCPAP (FiO2 25%), HR 145 bpm, non-invasive BP 55/23/36 mmHg. Central venous access was obtained, and continuous PGE1 infusion was started. A central arterial line was placed for invasive BP monitoring. Total parenteral nutrition, empirical antibiotic therapy and caffeine citrate were initiated in the first hour of life.

**Figure 4:**
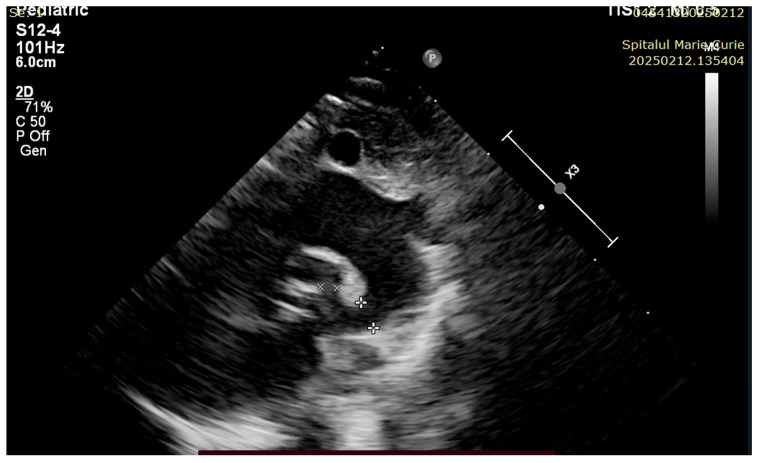
PDA—pulmonary end 0.22 mm; aortic end 0.37 mm.

**Figure 5:**
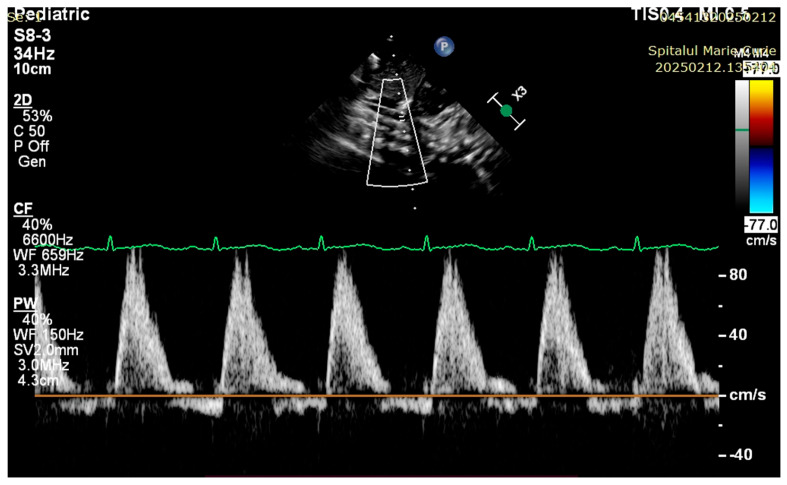
PW abdominal aorta. Diastolic “steal” secondary to non-restrictive PDA.

**Figure 6:**
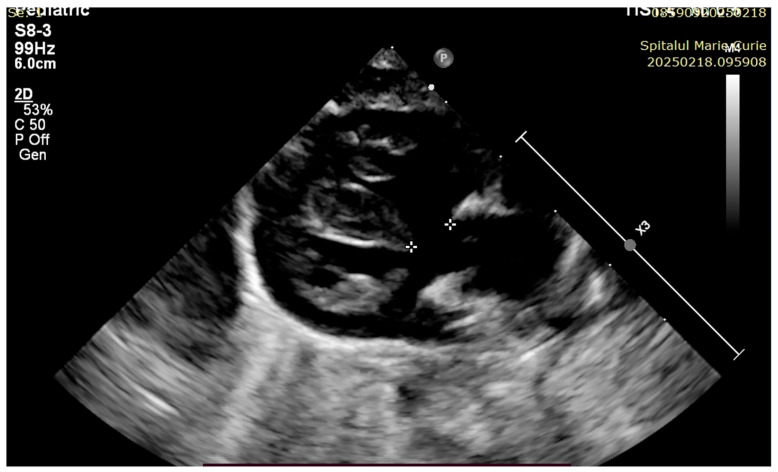
Ventricular septal defect and overriding aorta.

**Figure 7:**
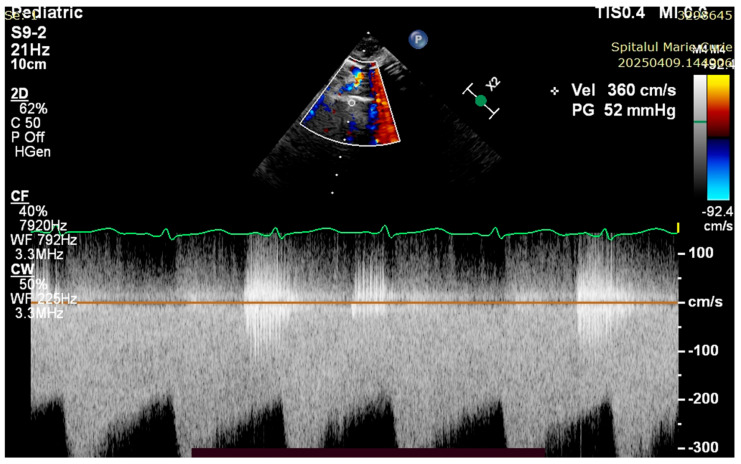
Severe limitation of anterograde blood flow via pulmonary artery. Complete RVOTO.

The patient was evaluated by a pediatric cardiologist with the following findings: PA with ventricular septal defect, the trunk and branches of the pulmonary artery are visualized, the interatrial septum with discontinuity in 1/3, with left–right shunt, DA 2.5 mm, with bidirectional flow (Figure 4, Figure 5, Figure 6 and Figure 7).

In the NICU, BP continued to drop, so continuous Dopamine infusion was started in doses up to 10 µg/kg/min, with a mean BP improvement. Vasopressor infusion was continued for 17 days. POCUS evaluations were performed every 3 days, adjusting the PGE1 infusion based on DA diameter, blood flow pattern, abdominal aortic flow and SpO2 levels (targeting 80–85%). The patient required a progressive increase in the dose of PGE1 up to 0.07 µg/kg/min in order to maintain an adequate PBF. Non-invasive mechanical ventilation was maintained for 55 days, in order to control SpO2, WOB and PaCO2, avoiding overdistension and hyperoxia. Systemic postnatal corticosteroids (>7 days of life) for bronchopulmonary dysplasia prophylaxis were added into the treatment, without significant improvement.

Based on the clinical (respiratory instability with apnea or increased need for ventilation support, lethargy) and laboratory (Leucocyte count > 20,000/mm^3^, CRP > 15 mg/L) findings, the patient was diagnosed with culture-negative late-onset sepsis, for which he received broad-spectrum antibiotic therapy for 14 days.

For optimal growth, the patient needed high energy intake, so total parenteral nutrition was initiated from the first day of life for 5 days. Enteral feeding was started from day 3 of life through a nasogastric tube with hypercaloric formula to reach 150 kcal/kg/day with high protein intake (up to 4 g/kg/day), because both human milk and pasteurized donor human milk were not available. NGT feedings were discontinued for 2 days, when diastolic “steal” was present (Figure 5), and restarted after decreasing the PGE1 infusion dose, and the POCUS evaluation revealed laminar flow in abdominal aorta.

On day 55 of life, the patient was transferred to a cardiovascular surgery center in order to perform modified BT shunt.

## 5. Discussions

Infants with ToF PA and complete RVOTO present a complex management challenge in the NICU. These infants often require multidisciplinary care involving the cardiology, neonatology and nutrition fields, particularly given their hemodynamic instability and unique physiological profile.

### 5.1. Clinical Stabilization and Hemodynamics

Immediate postnatal management focuses on ensuring adequate PBF via the DA. PGE1 remains the standard intervention to maintain ductal patency. However, the response can be variable depending on the ductal anatomy and the presence of pulmonary artery hypoplasia, as illustrated in our first case. In this context, POCUS has proven essential, allowing for the real-time monitoring of PDA size, blood flow direction and systemic circulation compromise, which is critical when pediatric cardiology expertise is not immediately available [13,14].

In cases where systemic hypotension coexists with hypoxia, a vasopressor strategy such as the use of norepinephrine or vasopressin may be needed to maintain SVR without increasing pulmonary vascular resistance (PVR) [28]. iNO may be considered when PGE1 fails, especially in the presence of elevated PVR or overlapping PPHN [38].

### 5.2. Respiratory and Ventilatory Considerations

A stepwise, physiology-guided approach should be employed, prioritizing lung protective strategies and spontaneous breathing when feasible. Spontaneous efforts can reduce intrathoracic pressure and thereby PBF through the ductal pathway [39,41]. Overventilation, especially with high PEEP, may lead to overdistension, increased PVR and subsequent reductions in pulmonary perfusion—thus undermining oxygenation and hemodynamics [39].

### 5.3. Nutritional Strategy

Nutritional management is an essential, yet often underemphasized, aspect of the care of newborns with ToF with RVOTO. These neonates have high metabolic demands and limited capacity to feed orally due to fatigue, respiratory distress or compromised perfusion. Enteral nutrition, particularly with human milk, should be initiated early if hemodynamic stability allows for it [42].

However, mesenteric perfusion may be impaired due to ductal steal phenomena, especially in patients with a large PDA and reversed diastolic flow in the abdominal aorta. In such cases, our practice involves temporarily interrupting enteral feeds, followed by cautious reintroduction guided by POCUS and clinical markers [43].

Parenteral nutrition remains a cornerstone of support during the early days of life, particularly in preterm or critically ill neonates, to meet the high protein (up to 4 g/kg/day) and energy (up to 150 kcal/kg/day) requirements essential for surgical readiness [43]. Hypercaloric formula may be required in the absence of human milk or to augment weight gain when feeding volume is limited.

### 5.4. Surgical Timing and Outcome Considerations

The timing of surgical intervention, whether palliative or corrective, significantly impacts long-term outcomes. Factors such as birth weight, hemodynamic stability and center-specific expertise influence the decision for early complete repair versus staged palliation (e.g., PDA stenting or modified BT shunt) [25,44].

Infants under 2.5 kg carry higher surgical risk, making early growth optimization a key goal of preoperative management [45]. As illustrated in our second case, meticulous hemodynamic monitoring, nutritional support and respiratory management facilitated stabilization and successful transfer for surgery.

### 5.5. Need for Standardization and Training

There is an urgent need for standardized care protocols, including ventilatory strategies, PGE1 dosing, feeding guidelines and the timing of surgical consultation. Training neonatologists in cardiac-focused POCUS and ensuring safe neonatal transport systems are essential, especially in resource-limited or geographically dispersed settings. Further research and multicenter registries are needed to define the best practices for this high-risk neonatal subgroup.

### 5.6. Article Limitations

This article is based on case reports and management strategies from a single tertiary NICU center, which may limit the generalizability of the findings to other institutions with differing resources, protocols or surgical timelines. The presented cases focus exclusively on preoperative management and short-term stabilization, without long-term follow-up on neurodevelopmental, nutritional or surgical outcomes. This restricts the ability to evaluate the full impact of the proposed management strategies on patient prognosis.

## 6. Conclusions

The preoperative management of ToF remains heterogenous among individual centers. The current literature is scarce, and clinicians must adapt the treatment following the physiology of the disease. In the absence of established guidelines for this specific subgroup of CHD patients, we propose a graduated approach guided by expert opinion and institutional experience. In most severe cases, especially those with PA or the absence of the ductus arteriosus with a severe limitation of the PBF, surgical approaches remain the main drivers of the survival rate and psychomotor outcomes. The time from diagnosis to surgery is related to adverse outcomes (neurodevelopmental delay, heart failure and arrythmias) secondary to prolonged chronic hypoxia [48]. In order to reduce the preoperative duration, there is an urgent need to train more surgeons with expertise in critical cardiac conditions for the pediatric population. Also, there is a need for expertise in the safe transportation of critical care infants to local surgical centers or high-income countries.

## Figures and Tables

**Table 1:** Recommendations for the delivery room stabilization of infants.

Category	Standard	Intervention
Thermal control	Monitor and record infant temperature after birth Maintain temperature between 36.5 and 37.5 °C Avoid hypothermia (<36.5 °C) or hyperthermia (>38.5 °C)	Environment temperature between 23 and 25 °C. Skin-to-skin care if no resuscitation is needed. Using radiant warmer with servo-control if resuscitation is performed.
Umbilical cord	Delay cord clamping for at least 60 s if feasible	If there is no need for resuscitation, clamping should be delayed after lung aeration. If adequate thermal care and resuscitation is possible, these interventions can be performed with intact cord. Consider milking cord if delayed cord clamping is not feasible in infants > 28 weeks.
Initial assessment	Assess heart rate, tone and breathing at birth and every 30 s	Initial assessment can be performed via observation and auscultation.
Initial steps	Positioning Drying and stimulating Suctioning	Place infant’s head in neutral position. Face is horizontal. Neck is neither flexed nor extended. Start drying and tactilely stimulating infant after birth. Perform suctioning only if visible obstruction is present. Perform suctioning before PPV is delivered.
PPV	Start PPV if heart rate remains <100 bpm or if infant is apneic, gasping or not breathing effectively Assess chest movement and heart rate Use pulse oximetry to assess SpO2 and heart rate	PPV should be delivered using “T” piece resuscitator. Initial peak pressure 30 cm H2O for term infants and 25 cm H2O for preterm infants. Use PEEP 6–8 cm H2O, if possible. FiO2 21% for term infants and 21–30% for preterm infants. Adjust FiO2 accordingly with SpO2.
Alternate airway	Orotracheal intubation in delivery room Consider laryngeal mask in infants > 1500 g or when intubation is not possible	Consider alternative airway when adequate ventilation is not achieved after corrective steps are performed. Consider alternative airway when performing chest compressions.
Chest compressions	Consider chest compressions when heart rate is <60 bpm when good-quality ventilation is delivered	Use synchronous technique. Provide chest compression to ventilation 3:1. Increase FiO2 to 100%.
Medication	Consider intravenous access Intraosseous access can be alternative for emergency access	iv Adrenaline 10–30 micrograms/kg if heart rate remains <60 bpm after 30 s of chest compressions and ventilation. Consider iv bolus glucose 250 mg/kg (2.5 mL/kg of glucose 10%) in prolonged resuscitation. Consider volume replacement (10 mL/kg O Rh negative blood or isotonic crystalloid) if volume depletion or unresponsive shock is suspected.

PPV—positive pressure ventilation; bpm—beats per minute. Table 1 was adapted from Madar et al. [11].

**Table 2:** Ventilatory management. Initial set-up and adjustment [41].

Parameter	Set-Up and Adjustment	Rationale
Tidal volume	V_T_ 4.5–5.5 mL/kg Titrate V_T_ in steps of 0.5 mL/kg based on blood gas analysis and work of breathing	Maintain PaCO2 within normal ranges Tachypnea may develop when lower tidal volumes are used If Ph is normal, moderate hypercapnia (PaCO2 45–60 mmHg) may be tolerated If Ph is normal, mild hypocapnia (PaCO2 30–35 mmHg) may be tolerated
Pressure support	Set pressure support to deliver at least 4 mL/kg Titrate in steps of 1 cm H2O	Insufficient pressure support may deliver a lower tidal volume that leads to the ventilation of dead space Increase the level of support to avoid distress and maintain a normal respiratory rate
PEEP	Set initial PEEP based on SpO2 and FiO2 4–6 cm H2O may be a good start Titrate in steps of 0.5 cm H2O	Optimal PEEP promotes good lung recruitment and prevents overdistension Optimal PEEP allows for lower FiO2 Overdistension increases PVR, limiting PBF
Pmax	Set Pmax above 25–30% of working PIP	Alerts the clinician about changes in patient status Alerts the clinician about leaks or endotracheal tube displacement
Respiratory rate	Aim for a moderate rate of 25–35 breaths/min Adjust in steps of 3–5 breaths/min	Allows the infant to trigger spontaneous breaths while maintaining the mandatory rate for lung recruitment Adjust to maintain PH < 7.35 when the weaning process is started; PH is more responsible for respiratory drive than PCO2
Inspiratory time	Set initial inspiratory time based on the pulmonary disease and gestational age Adjust in steps of 0.1 s based on the ventilatory waveforms	Lower inspiratory times are needed in homogenous pulmonary disease (e.g., RDS) Longer inspiratory times may be needed in heterogenous pulmonary disease (e.g., BPD, MAS) A short inspiratory time might deliver insufficient volume A long inspiratory time might result in insufficient expiratory time and air trapping

V_T_—tidal volume; PIP—positive inspiratory pressure; PEEP—positive end expiratory pressure; BPD—bronchopulmonary dysplasia; RDS—respiratory distress syndrome; MAS—meconium aspiration syndrome.

## Data Availability

The original contributions presented in this study are included in the article. Further inquiries can be directed to the corresponding author.
